# Coupled electro-thermal field in a high current electrolysis cell or liquid metal batteries

**DOI:** 10.1098/rsos.171309

**Published:** 2018-02-14

**Authors:** Ze Sun, Liwei Cai, Haiou Ni, Gui-Min Lu, Jian-Guo Yu

**Affiliations:** 1 School of Resources and Environmental Engineering, East China University of Science and Technology, Shanghai 200237, People's Republic of China; 2 National Engineering Research Center for Integrated Utilization of Salt Lake Resource, East China University of Science and Technology, Shanghai 200237, People's Republic of China

**Keywords:** electric field, thermal field, numerical simulation

## Abstract

Coupled electro-thermal field exists widely in chemical batteries and electrolysis industry. In this study, a three-dimensional numerical model, which is based on the finite-element software ANSYS, has been built to simulate the electro-thermal field in a magnesium electrolysis cell. The adjustment of the relative position of the anode and cathode can change the energy consumption of the magnesium electrolysis process significantly. Besides, the current intensity has a nonlinear effect on heat balance, and the effects of heat transfer coefficients, electrolysis and air temperature on the heat balance have been released to maintain the thermal stability in a magnesium electrolysis cell. The relationship between structure as well as process parameters and electro-thermal field has been obtained and the simulation results can provide experience for the scale-up design in liquid metal batteries.

## 1. Introduction

With the large-scale deployment of renewable energy sources, massive and cheap electricity storage becomes indispensable because the major part of renewable electricity generation (solar, wind) is inherently fluctuating. Liquid metal batteries (LMBs) proposed by professor Sadoway and co-authors [1] are currently discussed as a means to provide economic grid-scale energy storage. The liquid metal battery is high-temperature (700°C) magnesium–antimony (Mg||Sb) comprising a negative electrode of Mg, a molten salt electrolyte (MgCl_2_–KCl–NaCl), and a positive electrode of Sb. The original liquid battery begins with advances resulting in the development of the three-liquid-layer Hoopes cell at the Aluminum Company of America (Alcoa) in the 1920s for the electrolytic production of high-purity aluminium [2]. But actually, the other original liquid battery is the magnesium electrolysis cell. It releases liquid magnesium and chloride gas, compared with Hoopes cell which releases liquid aluminium and carbon dioxide. The characteristics of liquid metal batteries or magnesium electrolysis cells are nearly the same, such as electrolyte comprising alkaline and alkaline-earth metals, high temperature of 700°C, high current passing electrolyte, all metals being liquid and so on.

In any case, scalability is the key factor for new grid storage and easy to scale-up is one of the main fundamental assumptions of LMBs development. When LMB scales to large applications, it will encounter the same problem of coupled electro-thermal field in the same cell like magnesium electrolysis. The study of thermal field, electric field, flow field and magnetic field of LMBs is crucial to making this technology into commercialization. Shen & Zikanov [3] and Köllner *et al.* [4] have studied the thermal convection of small-scale prototypes by numerical simulation method. They found that it is necessary to develop a comprehensive model that describes the coupled physical phenomena of LMBs. Wang & Wang [5] compared the temperature distribution under various current densities and different battery electrolyte sizes, and the analysis is based on the finite-element numerical methods with a three-dimensional thermal simulation model of LMB. Weber *et al.* [6,7] and Stefani *et al.* [8] investigated the Tayler instability (TI) of LMBs by the simulation of electro-magnetic field and flow field. The effect of Tayler instability has been analysed and four methods have been developed to tame this effect. Weber *et al.* [9] and his colleagues also did the work on sloshing instability and electrolyte layer rupture by direct numerical simulation of flow field in LMBs, which is already known from aluminium reduction cells. However, there are few works focused on the electro-thermal field in LMBs, which is important to the scale-up design of the battery. Therefore, the investigations of electro-thermal field in magnesium electrolysis cells (high temperature and high current density) are necessary, and the results can provide experiences for the development of LMBs.

Many factors can influence the coupled electro-thermal field in a magnesium electrolysis cell, such as cell structure, operation parameters and physical field distribution [10,11]. There are six physical fields in the magnesium electrolysis cell, i.e. electric field, thermal field, magnetic field, concentration field, flow field and stress field [12]. The electric field is the energy source of the electrolysis cell, which is also the origin of other fields. First of all, strong current passes the cell and generates Joule heat, which is the reason why magnetic field and thermal field exists. Then, with the effect of electrolysis, chlorine precipitates at the anode and floats outside, which results in electrolyte flow and then generates flow field. The flow and diffusion of electrolyte cause concentration difference in the cell, so the concentration field cannot be ignored. Besides, the thermal field can influence the structure of the cell because of the heat stress, then the stress field needs to be considered. The complex coupling relationship between the six physical fields will have a direct impact on the current efficiency, energy consumption and lifespan of the magnesium electrolysis cell. These conditions have to be considered carefully at the beginning of designing a magnesium electrolysis cell. Among these factors, the electro-thermal field distribution is closely related to the heat balance, the energy consumption and lifespan of the cell [13]. Therefore, the electro-thermal field is significant in improving production efficiency. The electro-thermal simulation model is based on structural parameters, operation parameters (current intensity, electrolysis temperature, ambient temperature, anode and cathode distance (ACD), cell voltage, etc.), material properties and heat dissipation conditions. Apparently, the mathematical modelling tool is the best choice to study the model [14].

In the last decades, a lot of work has been done in the designing of an aluminium reduction cell with the development of computer technology. In the beginning, the inner space [15] and the thermal field distribution [16] of aluminium reduction cell were simulated by one-dimensional model. The one-dimension model is simple but inaccurate. Then with the promotion of the calculation speed, two-dimensional or three-dimensional model became the first choice to researchers. By using two-dimensional model, their research mainly focused on the problems of coupled electro-thermal equation [17,18], thermal field simulation [11], heat transfer method (convection and radiation) [19] and model partition [20]. In recent years, plenty of three-dimensional models of aluminium reduction cell have been constructed with the help of finite-element software ANSYS [21,22]. Besides, the three-dimensional models have been adopted in the studies of the electro-thermal field [23], electro-magnetic field [24], thermo-electro-mechanical model [25] and magneto-hydro-dynamic model [26,27]. The new three-dimensional models of electro-thermal field coupling, which were reported since 2016, have faster computing speed, better accuracy and wider application [28,29]. The studies of magnesium electrolysis cell are fewer than those of aluminium reduction cell relatively. Shcherbinin *et al.* [30] investigated the heat balance in a magnesium electrolysis cell by the mathematical simulation of the three-dimensional thermal field and electric field. Sun *et al.* [31–33] studied the flow field and the electric field in a magnesium electrolysis cell by simulation, where the effect of the electro-magnetic field of the cell was considered. Liu *et al.* [34] and his colleagues studied the scale-up design of a 300 kA magnesium electrolysis cell using a three-dimensional thermo-electric mathematical model, and the results can be referenced in the scale-up of magnesium electrolysis cells with a high current intensity.

In this paper, the study of magnesium electrolysis cell is mainly focused on the thermal balance and energy saving under normal production circumstances. With the help of finite-element software ANSYS, the effects of cell structure and operation parameters (current intensity, electrolysis temperature, ambient temperature, ACD, cell voltage, etc.) on thermal balance of magnesium electrolysis cell have been simulated in a three-dimensional model. Through analysing the thermal field distribution in the anode, cathode and other parts of the actual 120 kA magnesium electrolysis cell, the results of the study are useful to designing and optimizing magnesium electrolysis cells and benefit to the scale-up design of LMBs to a certain extent.

## 2. Electric and thermal coupling analysis model

### 2.1. Physical model

**Figure 1.**
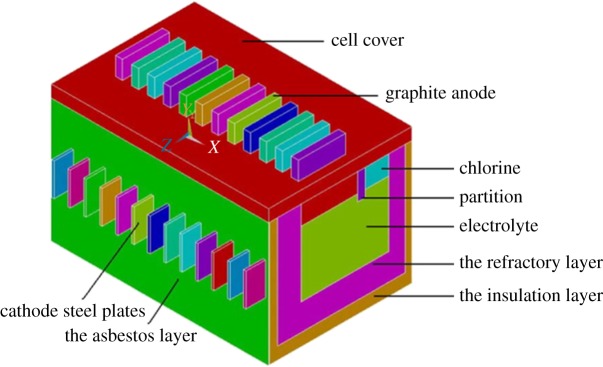
Schematic of magnesium electrolysis cell.

The magnesium electrolysis cell model should be simplified to meet the basic requirements of simulation because of its complicated structure and big size. The physical model in this paper includes graphite anodes, steel cathodes, electrolyte, firebricks, insulation bricks as well as cell shells. The other parts of less impact are not considered in the simulation. According to the symmetry of the long axis, the model is calculated by taking the one-half size of the full cell (as shown in figure 1).

In order to facilitate the finite simulated computation of the magnesium electrolysis cell, some assumptions are made as follows:

(1) In the magnesium electrolysis cell, the electric field and thermal field are assumed to be steady.
(2) The temperature of the electrolyte and magnesium are equal to the electrolysis temperature.
(3) The influences between the two half parts of the cell are ignored.
(4) The heat loss of the bus bar part is ignored.
(5) The round chamfer is treated as a right angle.

### 2.2. Mathematical model

The heat of the magnesium electrolysis cell is derived from the Joule heat generated by the current, and the Joule heat is dissipated from the outer surface of the cell to the outer space by convection and radiation. For the complex problem of coupling of cell convection and heat transfer, there are two modelling solutions at present: one is to use partition modelling and calculation, and the other is overall modelling and coupling solution. For the calculation object like magnesium electrolysis cell with complex cell shape, coupled with complex boundaries, if the partition model is adopted, the simulation results will vary greatly with the actual ones. Therefore, this study uses overall modelling and coupling solution.

#### 2.2.1. Control equation

In the computation model with electric and thermal field coupled, the electricity and heat transfer within the magnesium electrolysis cell are subject to the Laplace equation (equation 2.1) and the Poisson equation (equation 2.2) with internal heat source, respectively. The resistivity of the material varies with the temperature considering the influence of temperature,

2.1 $$\frac{\partial }{\partial x}\left(\frac{1}{\rho x}\frac{\partial V}{x}\right)+\frac{\partial }{\partial y}\left(\frac{1}{\rho y}\frac{\partial V}{y}\right)+\frac{\partial }{\partial z}\left(\frac{1}{\rho z}\frac{\partial V}{z}\right)=\frac{\partial V}{\partial \tau },$$

where *ρ*x, *ρ*y and *ρ*z are the resistivity of the material change with the temperature in three dimensions, respectively, Ω m. *V* is the electric potential (V) and *τ* the time (s); when *τ* is steady, ∂*V*/∂*Vτ* = 0.

According to heat transfer theory, the heat transfer of the constituent components in the magnesium electrolysis cell is subject to the unstable heat transfer control equation (equation 2.2) with an internal heat source. The equation in the three-dimensional model is

2.2 $$\frac{\partial }{\partial x}\left(k_{x}\frac{\partial T}{x}\right)+\frac{\partial }{\partial y}\left(k_{y}\frac{\partial T}{y}\right)+\frac{\partial }{\partial z}\left(k_{z}\frac{\partial T}{z}\right)+q_{s}=\rho c\frac{\partial T}{\partial \tau },$$

where *k_x_*, *k_y_* and *k_z_* represent the coefficient of heat conductivity (W m^−1^ °C^−1^). *T* is the temperature (K), *q_s_* the heat intensity, *ρ* the material density (kg m^−3^) and *c* the specific heat (W m^−1^ °C^−1^).

#### 2.2.2. Boundary conditions

The boundary conditions of electricity conduction equation are expressed as follows:

(1) Take the cathode plate surface as zero potential.
(2) Voltage degree of freedom is applied to the coupling node on the surface of the anode head, and then load the current of the electrolysis cell at either node.

The boundary conditions of heat conduction equation are expressed as follows:

(1) The temperature of the electrolyte is constant and equal to the electrolysis temperature.
(2) The temperature around the magnesium electrolysis cell is constant and equals to the actual temperature of the workshop.
(3) The heat dissipation coefficient of the outer surface of the electrolysis cell, the heat transfer coefficient between the electrolyte and the side cell surface, the heat transfer coefficient between the electrolyte and the cathode and anode surfaces as well as the heat dissipation coefficient of the cathode and anode will be obtained through the empirical formula which can be found in [35,36].
(4) When applying heat convection and transfer boundary conditions to the interface of the electrolysis cell and external surface, the heat transfer coefficient shall include the influence of radiation heat transfer.

#### 2.2.3. Finite-element analysis of the electro-thermal field model

For the above electro-thermal field model, the Galerkin method with weighted residual approach is used for the solution of finite-element model. The steady thermal field of the conductive part of the electrolysis cell can be expressed as

2.3 $$J[T(x,y,z)]=\lambda_{x}\frac{\partial^{2}T}{\partial x^{2}}+\lambda_{y}\frac{\partial^{2}T}{\partial y^{2}}+\lambda_{z}\frac{\partial^{2}T}{\partial z^{2}}+q_{\mathrm{vol}}=0,$$

where *λ*_x_, *λ*_y_ and *λ*_z_ are the heat conduction coefficient in the three dimensions, *x*, *y* and *z*; *q*_vol_ is the Joule heat of the control unit.

2.4 $$q_{\mathrm{vol}}=\sigma_{x}{\left(V\right)}^{2}\frac{yz}{x}+\sigma_{y}{\left(V\right)}^{2}\frac{xz}{y}+\sigma_{z}{\left(V\right)}^{2}\frac{xy}{z}.$$

The trial function can be expressed as

2.5 $$\bar{T}(x,y,z)=\bar{T}(x,y,z,T_{1},T_{2},KT_{n}),$$

where *T*_1_, *T*_2_ and *KT_n_* are undetermined coefficients.

Taking equation (2.5) into equation (2.3), the result obtained through weighted residual method is as follows:

2.6 $$\underset{\;D}{\int \int \int }W_{x}\;(\lambda_{x}\frac{\partial^{2}\bar{T}}{\partial x^{2}}+\lambda_{y}\frac{\partial^{2}\bar{T}}{\partial y^{2}}+\lambda_{z}\frac{\partial^{2}\bar{T}}{\partial z^{2}}+q_{\mathrm{vol}})\;\text{d}x\text{d}y\text{d}z=0.$$

According to the definition of weighted functions by the Galerkin method, weighted function *W_s_* can be expressed as:

2.7 $$W_{s}=\frac{\partial \bar{T}}{\partial T_{s}}{{,}_{}}_{}s=1,2,Kn.$$

Owing to the complexity of analysing the temperature field of the space, it is relatively difficult to find the *T*(*x*, *y*, *z*) satisfying the boundary conditions. And in order to meet the boundary conditions, the Gaussian formula can be used to connect the volume of the simulation area with the surface integral of the boundary. The following equation can be obtained through transformation:

2.8 $$\begin{array}{rl} \frac{\partial J}{\partial T_{S}} & =\underset{\;D}{\int \int \int }\left(\lambda_{X}\left(\frac{\partial W_{s}}{\partial x}\frac{\partial T}{\partial x}\right)+\lambda_{y}\left(\frac{\partial W_{s}}{\partial y}\frac{\partial T}{\partial y}\right)+\lambda_{z}\left(\frac{\partial W_{s}}{\partial z}\frac{\partial T}{\partial z}\right)-q_{\mathrm{vol}}W_{s}\right)\text{d}x\text{d}y\text{d}z\\ & \;-\oint_{\sum }\left(\lambda_{x}W_{s}\frac{\partial T}{\partial x}\cos\alpha +\lambda_{y}W_{s}\frac{\partial T}{\partial y}\cos\beta +\lambda_{z}W_{s}\frac{\partial T}{\partial z}\cos\gamma \right)\text{d}S=0,s.1,2\ldots n, \end{array}$$

where cos*α*, cos*β* and cos*γ* represent the cosine in the three dimensions *x*, *y* and *z*, respectively.

Equation (2.8) defines the whole solving area. Direct solving is quite difficult, so computation of equation (2.8) in the whole area is not adopted. On the contrary, the calculation of each grid unit in each part is conducted, and then they will be combined together to form whole linear equations to solve the question. If the area is divided into *E* units and *n* nodes, then the temperature *T*(*x*, *y*, *z*) will split into the undetermined temperature of the nodes like *T*_1_, *T*_2_ and *T*_3_.

2.9 $$\frac{\partial J}{\partial T_{S}}=\sum_{e=1}^{E}\frac{\partial J_{e}}{\partial T_{s}}=\frac{\partial J_{1}}{\partial T_{S}}+\frac{\partial J_{2}}{\partial T_{S}}+\cdots +\frac{\partial J_{E}}{\partial T_{S}}=0,s=1,2,Kn.$$

The combined equations can be written into the matrix form:

2.10 $$\begin{array}{rl} \left[k][T\right] & =[P]\\ \left[\begin{array}{cccc} k_{11} & k_{12} & \Lambda & k_{1n}\\ k_{21} & k_{22} & \Lambda & k_{2n}\\ & & \Lambda & \\ k_{n1} & k_{n2} & \Lambda & k_{nn} \end{array}\right]\;\left[\begin{array}{c} T_{1}\\ T_{2}\\ M\\ T_{n} \end{array}\right] & =\left[\begin{array}{c} P_{1}\\ P_{2}\\ M\\ P_{n} \end{array}\right] \end{array}$$

In equation (2.10), *k* represents the temperature stiffness matrix; [*T*] = [*T*_1_, *T*_2_, …., *T_n_*]; *P* represents the temperature load matrix, the temperature of each node of the magnesium electrolysis cell can be calculated through the above equation.

### 2.3. Finite-element model analysis

**Figure 2.**
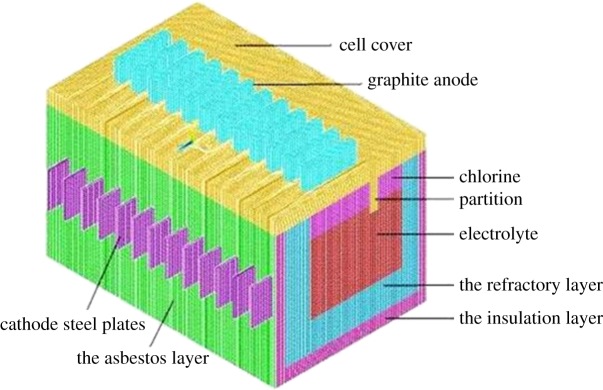
Sketch of half magnesium electrolysis cell mesh.

The finite-element model of magnesium electrolysis cell is established according to the above methods, and the cell type includes conductive part and un-conductive part. The conductive part adopts unit SOLID69 to analyse the electro-thermal field, while the un-conductive part adopts unit SOLID70 for thermal analysis. In order to get accurate calculation results, hexahedron grid is used to divide the cell. The size of each grid is 0.04 m. There are nine materials used in the electrolysis cell. The characteristics of these materials vary with temperature, so the nonlinear processing is used to treat these materials. The specific finite-element model is shown in figure 2.

The number of hexahedron grids of the finite-element model adopted in this study is 600 000–2 000 000, and accurate calculation results can be obtained if the hexahedron grids are within the number.

### 2.4. Validation and thermal equilibrium of the magnesium electrolysis cell

In this paper, a 120 kA magnesium electrolysis cell in China is set as an example to illustrate the thermoelectric coupling simulation calculation method. According to the above description, a three-dimensional half cell model is used here for the simulation of the electro-thermal field coupling in the magnesium electrolysis cell.

#### 2.4.1. Modelling and solving

For the cathode, anode and electrolyte, coupling unit SOLID69 with two degrees of freedom (temperature and voltage) will be adopted, while the others will use SOLID70 unit with only one degree of freedom (temperature). The outer convection heat transfer part will use SURF152 unit to exert boundary conditions and calculate the amount of dissipated heat. The material properties of the magnesium electrolysis cell are nonlinear in the electrolysis process. Therefore, MPDATA order will be used to define the materials properties so as to reflect the distribution of the internal temperature field of electrolysis cell. Tables 1–3 show the structure parameters and materials properties of different cell types.

**Table 1.** Main structure parameters of 120 kA magnesium electrolysis cell.

direction	refractory layer	insulation layer	asbestos plate	steel shell
−*x*/m	0.23	0.115	0.01	0.01
−*y*/m	0.566	0.264	0.01	0.01
−*z*/m	0.345	0.14	0.01	0.01
+*z*/m	0.495	0.18	0.01	0.01

**Table 2.** Materials resistivity with temperature change for magnesium electrolysis cell [35].

material (Ω · m)	100°C	200°C	300°C	400°C	500°C	600°C	700°C	800°C
anode	8.17 × 10^−6^	7.68 × 10^−6^	7.38 × 10^−6^	7.24 × 10^−6^	7.22 × 10^−6^	7.28 × 10^−6^	7.38 × 10^−6^	7.51 × 10^−6^
cathode	2.10 × 10^−7^	2.85 × 10^−7^	3.60 × 10^−7^	4.35 × 10^−7^	5.10 × 10^−7^	5.85 × 10^−7^	6.60 × 10^−7^	7.35 × 10^−7^
electrolyte	4.50 × 10^−3^	—	—	—	—	—	—	—

**Table 3.** Materials conductivity with temperature change for magnesium electrolysis cell [35].

material (w m^−1^ k^−1^)	100°C	200°C	300°C	400°C	500°C	600°C	700°C	800°C
anode	120	120	120	120	120	120	120	120
cathode	57.000	53.000	49.300	45.500	41.000	37.000	33.000	28.500
electrolyte	50	—	—	—	—	—	—	—
refractory layer	1.2065	1.2065	1.2191	1.243	1.2978	1.35345	1.4224	1.56765
insulation layer	0.1768	0.2082	0.2396	0.271	0.3024	0.3338	0.3652	0.3966
asbestos plate	0.174	0.181	0.189	0.196	0.204	0.211	0.219	0.226
cell cover	1.03	1.03	1.03	1.03	1.03	1.05	1.05	1.05
chlorine	0.0321	0.0393	0.046	0.0521	0.0574	0.0622	0.0671	0.0781

**Figure 3.**
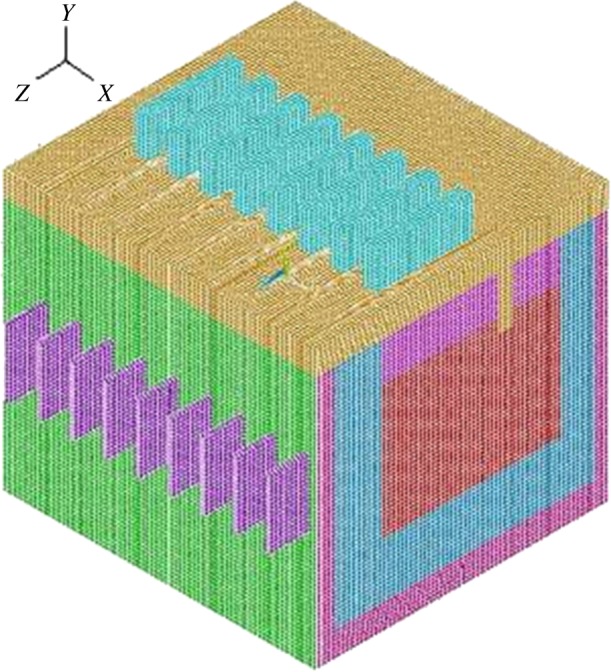
Computational domains for 120 kA magnesium electrolysis cell.

The model includes anode, cathode, electrolyte, refractory layer, insulation layer, thermal-protective layer, cell cover and chlorine. The size of the grid unit is 0.04 m, and each part of the model uses hexahedron grid. The total number of grid is 668 889 with a calculating time of about 2200 s. The finite-element cell model is shown in figure 3.

The boundary conditions of the model can be expressed as follows:

(1) The environment temperature equals 25°C.
(2) The electrolysis temperature equals 700°C.
(3) The anode head loads 60 000 A current and sets the cathode outlet potential to 0.
(4) The heat convection transfer coefficient for each section is listed in table 4 [35,36].

**Table 4.** Convective heat transfer coefficients for120 kA magnesium electrolysis cell [35,36].

items	convective heat transfer coefficient (W m^−1^ K^−1^)
cathode	13.64
anode	23.08
the front longitudinal wall	11.37
the back longitudinal wall	11.94
cell bottom	9.64
internal cell	100
cell cover	17.81
end wall	10.99
chlorine	100

## 3. Results and discussion

**Figure 4.**
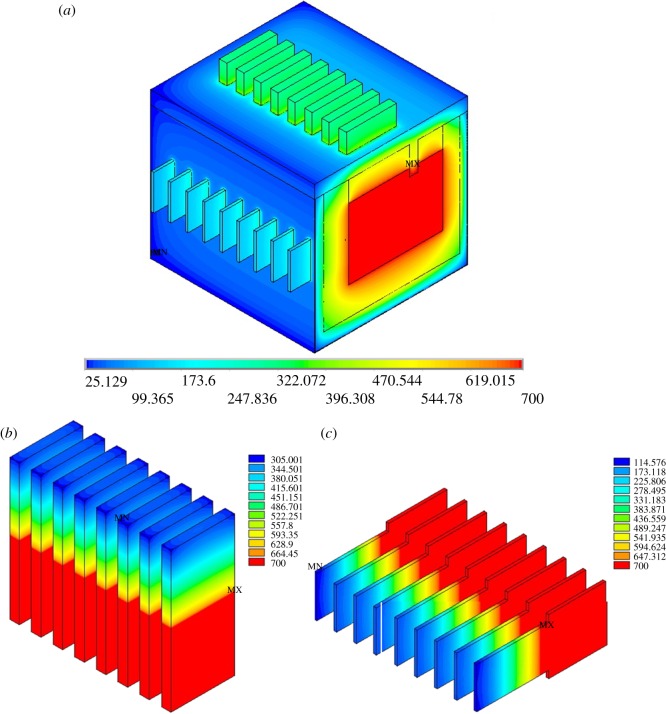
Temperature contour for 120 kA magnesium electrolysis cell.

Figure 4*a* shows the temperature isopleths of the entire electrolysis cell, while figure 4*b*,*c* is the temperature isopleths of anode and cathode. It can be seen that the highest temperature is in the electrolysis interval while the lowest temperature of the exterior wall closing to 25°C. The lowest temperature of the anode and cathode is 305°C and 114°C, respectively. The APDL which is developed from ANSYS is applied for average temperature estimation on anode head, cathode head, cell cover surface, front wall, back wall, end wall and cell bottom is 338.5°C, 99.2°C, 164.2°C, 61.0°C, 68.2°C, 72.8°C and 52.7°C, respectively. The simulated data are in good agreement with the actual empirical data. The most obvious feature is that the temperature distribution is uneven in every angle, which means that these parts are prone to thermal expansion and contraction, and it is also one of the most important factors affecting the lifespan of the cell.

**Figure 5.**
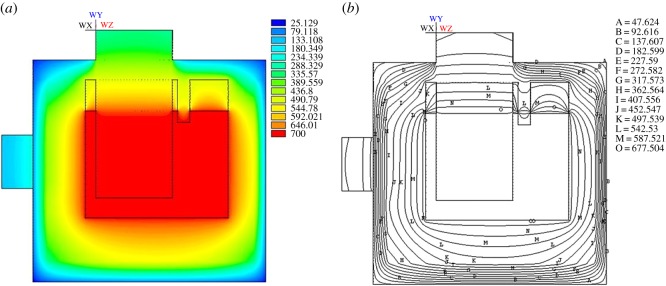
Temperature distribution of profile.

Figure 5*a,b* are the temperature isopleths for magnesium electrolysis cell in different directions. Figure 5*a* shows the heat transfer path in the cross section of the cell. The bottom of the cell has the lowest temperature, resulting from the relatively large heat transfer coefficient of the other parts, especially the anode head and the cathode. Owing to the heat resistance, the temperature of the electrolyte drops from 700°C to about 677°C at the cell boundary.

**Figure 6.**
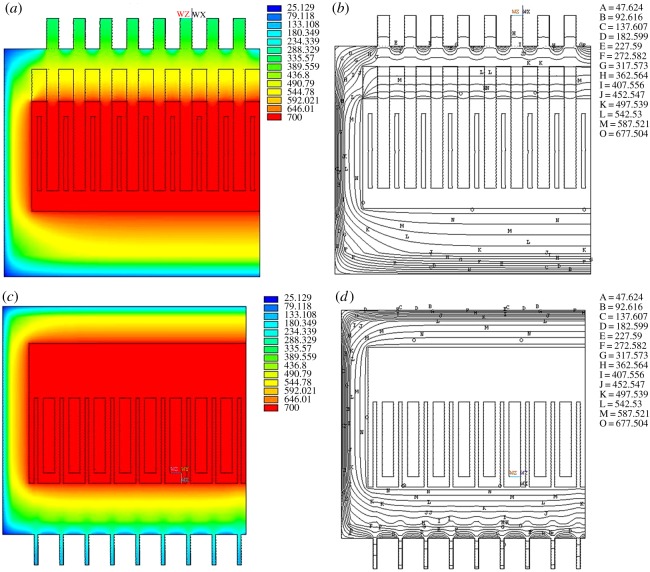
Front view and vertical view of temperature contour and isotherm.

Figure 6*a* examines the thermal field of the whole cell, and the heat dissipation of the anode head is much better than that of the cell cover. Figure 6*b* shows the temperature isopleths corresponding to figure 6*a*. It shows that the effect of heat preservation on the end wall and cell bottom is better than that of the cell cover and anode head. The range of the temperature variation is obviously smaller than other parts, especially when the temperature isopleth of the cell bottom reaches 497°C. Figure 6*c,d* show the vertical view of the temperature field and temperature isopleths, respectively. In figure 6*c*, the temperature near the cathode is higher than the rest of the surrounding parts because of the faster dissipation speed and bigger thermal conductivity coefficient of the cathode. Therefore, the temperature isopleth concaves inward. But when the temperature drops to about 317°C, the direction of temperature isopleth changes from concave to convex, because the heat dissipation near the cathode is great.

### 3.1. Energy equilibrium calculation and analysis

Table 5 shows the amount of heat produced and dissipated in each part of 120 kA magnesium electrolysis cell by using APDL language for post-processing.

**Table 5.** Heat balance for half magnesium electrolysis cell.

energy input			energy output		
items	kW	percentage	heat dissipating capacity	kW	percentage
anode	33.333	32.75	anode	60.629	59.40
cathode	8.397	8.25	cathode	11.154	10.93
electrolyte	60.043	59.00	cell cover	15.304	14.99
			back wall	3.673	3.59
			front wall	4.249	4.16
			end wall	4.335	4.25
			cell bottom	2.723	2.68
theoretical total energy	101.773	100	actual heat dissipating capacity	102.067	100
heat loss difference (%) 0.288					

The heat generated by the electrolyte accounts for 59% of the total, 32.75% for the anode and only 8.25% for the cathode. On the other hand, the dissipation heat of the anode head occupies 59.4% of the total, followed by 14.99% for the cell cover and 10.93% for the cathode. In general, the difference percentage of heat balance is less than 1%, which meets the requirement of actual production process. Therefore, the parameters of the anode, cell cover and cathode will be adjusted first if adjustment of heat balance is required.

Table 6 is a comparison of the thermal balance calculated by analytic method and ANSYS with the thermal analysis in actual production process, and ANSYS is better than analytic method. The heat loss difference reaches 3.75% by analytic method, and it is within the engineering calculation error of 10%. But it is 10 times of the result calculated by finite-element method. The energy loss of the two methods mainly differentiates in the heat dissipation capacity of the cathode.

**Table 6.** Comparison of energy consumption between finite-element method and analytic method.

energy consumption of analytic method			energy consumption of finite element		
heat dissipating capacity	kW	percentage	heat dissipating capacity	kW	percentage
anode	62.48	63.36	anode	60.629	59.40
cathode	6.325	6.41	cathode	11.154	10.93
cell cover	15.98	16.20	cell cover	15.304	14.99
longitudinal wall	7.580	7.68	back wall	3.673	3.59
			front wall	4.249	4.16
end wall	3.725	3.77	end wall	4.335	4.25
cell bottom	2.505	2.54	cell bottom	2.723	2.68
actual heat dissipating capacity	98.61	100	actual heat dissipating capacity	102.067	100
heat loss difference (%)	3.75		heat loss difference (%)	0.288	

### 3.2. The influence of structural parameter on heat equilibrium

The thermal balance of the magnesium electrolysis cell is an important criterion for measuring whether a magnesium electrolysis cell can work properly. The research will focus on the effects of the several factors on the thermal balance, which include current intensity, electrolysis temperature, ambient temperature, heat transfer coefficient and the distance between cathode and anode.

#### 3.2.1. The effect of current intensity on the thermal balance

The effects of current intensity of the different cell types, which contain maximum current value all over the world, have been studied in this paper. At the same time, it can provide a basis for designing a larger energy-saving magnesium electrolysis cell in the future. The paper examines the thermal balance of seven cell types including 120, 180, 240, 300, 360, 420 and 480 kA. The main research goal is whether it can be convenient or not to get a large magnesium electrolysis cell, after simplest amplification.

**Figure 7.**
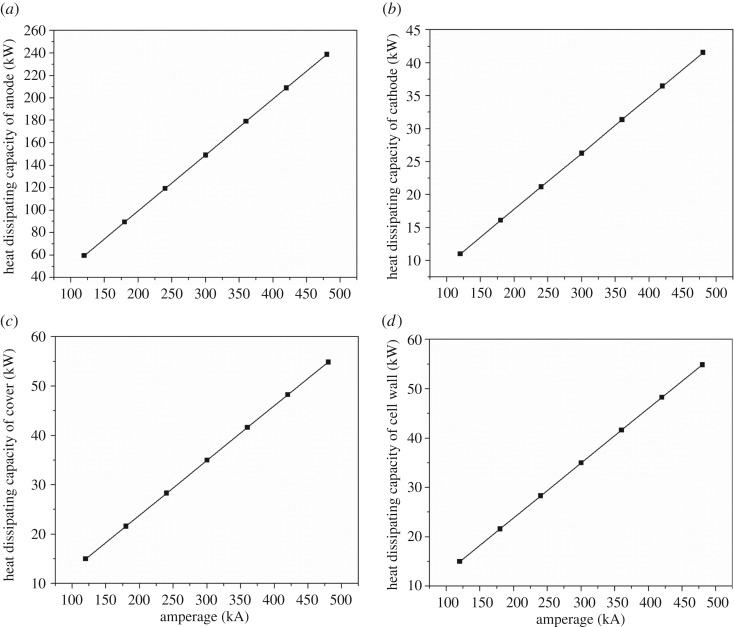
(*a*–*d*) The relationship between amperage and energy consumption.

As shown in figure 7, with the increase of the current intensity, the heat dissipating capacity of the anode, cathode, cell cover and cell shell increases linearly. The heat dissipation of anode increases from 59.6 to 238.7 kW, of cathode from 11 to 41.5 kW, of cell cover from 15 to 54.8 kW and of cell shell from 14.8 to 43.5 kW. The value of the heat dissipating capacity of each part in the cell remains unchanged. The proportion of anode, cathode, cell cover and cell shell accounts for about 59.3%, 11.0%, 14.9% and 14.8%, respectively.

**Figure 8.**
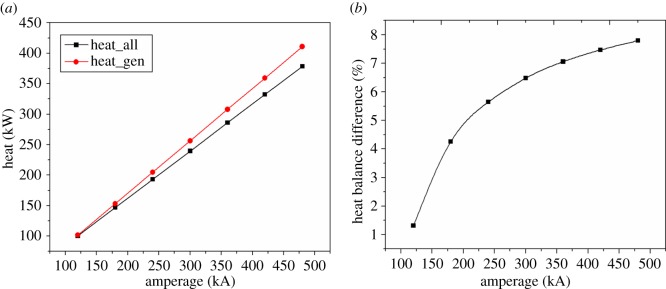
The relationship between heat balance difference and amperage.

As shown in figure 8, the thermal balance difference is increasing with the current intensity increasing, which means the simple parallel amplification can lead to thermal unbalance. As the magnesium electrolysis cell is not energy-saving in practical production, increasing the current intensity is not a suitable way to keep the heat balance.

Table 7 shows the temperature of each part of different current intensity calculated by APDL. As the current intensity increases, the temperature of anode head can be considered unchanged essentially, and the temperature increase rate of cathode is the largest, reached 9.12°C.

**Table 7.** Temperature of various parts in different amperage.

	temperature (°C)						
item	120 kA	180 kA	240 kA	300 kA	360 kA	420 kA	480 kA
anode head	343.29	343.49	343.53	343.60	343.67	343.68	343.71
cathode head	107.84	111.47	113.48	114.74	115.70	116.36	116.96
cell cover	172.39	174.36	174.89	175.41	175.84	176.15	176.39
back wall	70.69	71.87	72.38	72.75	73.042	73.24	73.39
front wall	77.69	78.69	79.23	79.57	79.80	79.97	80.11
end wall	82.27	82.27	82.27	82.27	82.27	82.27	82.27
cell bottom	62.46	63.43	63.95	64.27	64.47	64.65	64.78

#### 3.2.2. The effect of the electrolysis temperature on the heat balance

Keeping the electrolysis temperature steady is a basic condition to keep the electrochemical reaction. Besides, keeping the electrolysis temperature steady can prevent the electrolyte from cooling crystallization. Generally speaking, different electrolysis temperature levels have different effects on the heat balance of electrolysis cell. The current intensity of 120–300 kA of the magnesium electrolysis cell is mainly researched here; after that, the simulation research is also in the same range.

**Figure 9.**
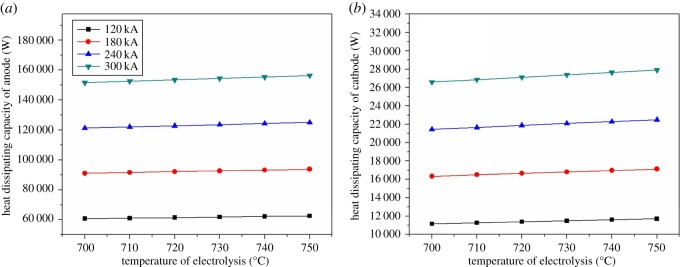
The relationship between electrolysis temperature and heat dissipating capacity of anode and cathode.

Figure 9 shows the relationship between electrolysis temperature and heat dissipating capacity of anode and cathode. In figure 9*a*, the heat dissipating capacity of the anode increases slightly with the increase of electrolysis temperature and current intensity. The electrolysis cell with different current intensity (120/180/240/300 kA) has different increase (1854.9/ 2791.6/ 3721.4/ 4652.2 W, respectively). In figure 9*b*, the heat dissipating capacity of the cathode increases with electrolysis temperature increasing. The maximum value of that growth appeared in the current intensity of 300 kA with the value of 1299.1 W.

**Figure 10.**
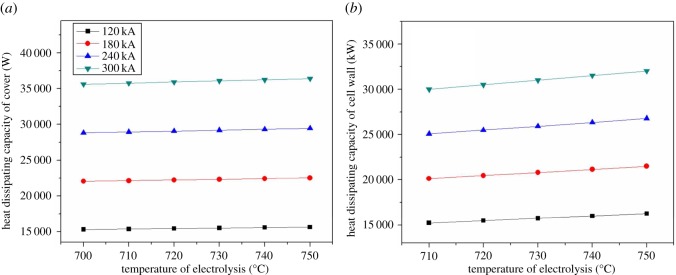
The relationship between electrolysis temperature and heat dissipating capacity of cover and shell.

In figure 10*a*, the heat dissipating capacity increases with the current intensity increasing. With the increase of electrolysis temperature, change of the heat dissipating capacity is smaller. The increasing rate of electrolysis temperature grows with the current intensity increasing, and the maximum of the increase rate is 777.6 W. In figure 10*b*, the increasing rate is obvious. The maximum increasing rate is 2531.7 W, and the minimum is 1287.0 W.

**Figure 11.**
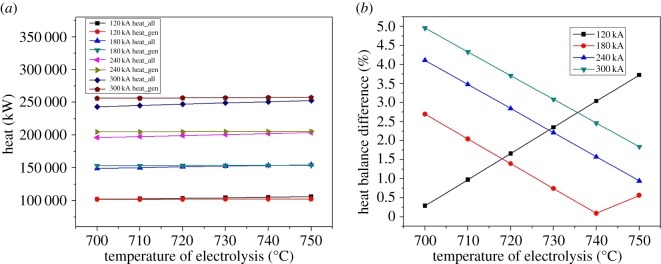
The relationship between heat balance difference and electrolysis temperature.

As shown in figure 11, in the same type of magnesium electrolysis cell, there is different heat balance temperature because of different current intensity. The balance electrolysis temperature of 120 kA is about 700°C, that of 180 kA is 740°C, and the others are more than 750°C. Figure 11*b* shows the heat balance deviation rate of different current intensity. The cell of 120 kA already reaches balance at 700°C. Therefore, with the increase of electrolysis temperature, the deviation rate increases in contrast. The line of 180 kA is exceptional, and there is an inflection point at 740°C. Before the inflection point, its trend agrees with the trend of 240 and 300 kA, but after the inflection point, its performance is similar to the trend of 120 kA.

#### 3.2.3. The effect of ambient temperature on the heat balance

The ambient temperature is the temperature of the production plant where the magnesium electrolysis cell is located. Generally speaking, ambient temperature of the electrolysis cell will change dramatically with season. However, in the traditional design, it commonly takes 25°C as ambient temperature, and the effect of the ambient temperature on heat balance has been ignored. Besides, the effects of regional difference and climate difference on heat balance have not been considered. Therefore, it is necessary and significant to research the ambient temperature of the magnesium electrolysis cell.

**Figure 12.**
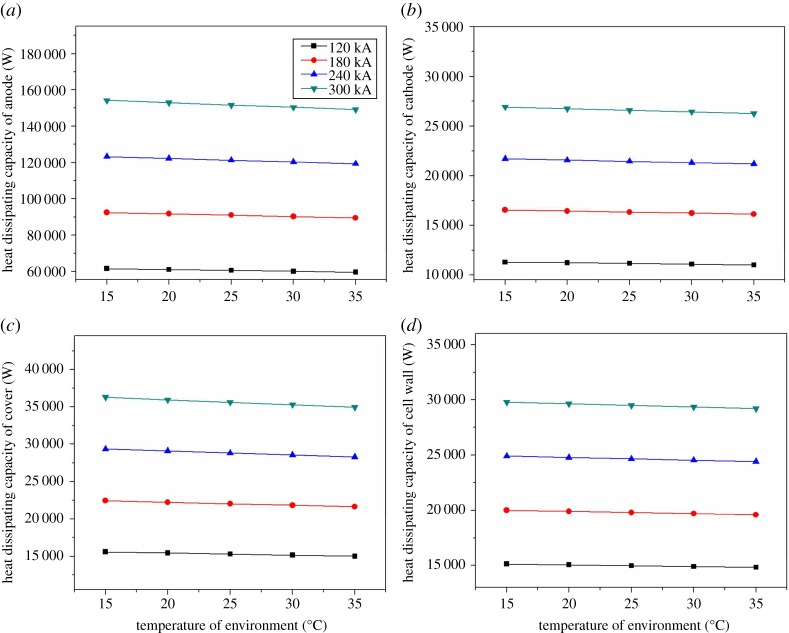
The relationship between environment temperature and various parts on heat balance.

As the figure 12*a–d* shows, the heat dissipating capacities of the different parts decrease with the increase of ambient temperature in different degrees. In figure 12*a*, heat dissipating of the anode increases with the current intensity. The heat loss of electrolysis cell 300 kA is 5011.9 W when the temperature is 15°C, and the maximum heat loss of 120 kA is 2003.9 W. In figure 12*b*, the heat changes slightly compared with figure 12*a*, and the maximum heat loss is about 658 W from 15°C to 35°C. Figure 12*c* shows the distribution of heat dissipation of cell cover in different current intensity, and the maximum point of the heat loss appears at 15°C when the current intensity is 300 kA, and its value is 1309.9 W. Figure 12*d* shows the heat loss of the cell shell, which is similar to the heat loss of cathode. The maximum heat loss is 592 W from 15°C to 35°C. In a word, the heat loss of the cell is about 7570.9 W in total with the increase of ambient temperature. The ambient temperature is a sensitive parameter to the electrolysis cell. Therefore, in order to optimize the design of the cell, the effect of ambient temperature must be taken into consideration.

#### 3.2.4. The effect of the anode heat transfer coefficient on heat balance

The anode is an essential part of the magnesium electrolysis cell. The heat dissipation of anode accounts for about 60% of the total. Therefore, the adjustment of the heat dissipation of the anode can effectively prevent the heat loss of the entire cell. In this study, changing the heat transfer coefficient of anode is the main method to improve the heat balance of the cell.

**Figure 13.**
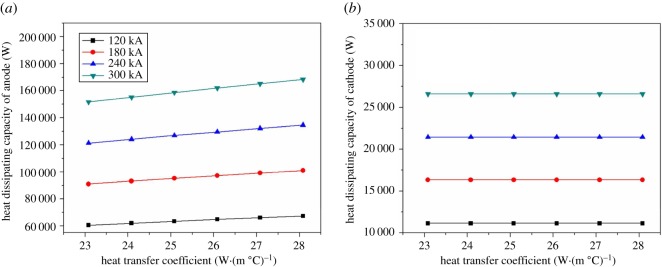
The relationship between anode heat transfer coefficient and the heat dissipating capacity of anode and cathode.

Figure 13*a* shows the heat dissipating capacity of anode head in different heat transfer coefficient under different current intensity. With the increase in heat transfer coefficient, the heat of the anode head shows an increasing trend. The heat transfer coefficient of the anode changing from 23 W·(m °C)^−1^ to 28 W·(m °C)^−1^, for the 120, 180, 240 and 300 kA electrolysis cell, makes the heat dissipating capacity of anode increasing by 6688.6, 10 030.1, 13 374.8 and 16 720.4 W respectively. Corresponding to the heat dissipating capacity of anode, its average growth rate is 9.93% by changing the heat transfer coefficient. Figure 13*b* shows the heat dissipating capacity of cathode. The heat loss is basically unchanged with the heat transfer coefficient of anode head changing, and the maximum of heat dissipating capacity is only 0.2 W, which can be neglected. Therefore, the change of the heat transfer coefficient of anode head has no impact on the heat dissipation capacity of cathode head.

**Figure 14.**
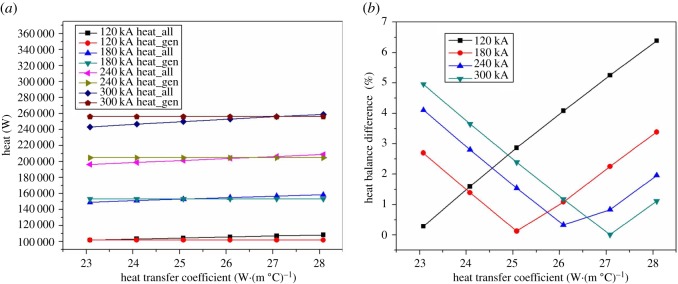
The relationship between anode heat transfer coefficient and heat balance difference.

As figure 14 shows, for electrolysis cells in all kinds of current intensity, the heat balance point changes with the heat transfer coefficient of anode. For 120 kA electrolysis cell, its balance point is at 23 W·(m °C)^−1^. Therefore, the heat balance difference increases with increasing heat transfer coefficient of anode head. Figure 14*b* shows the balance point more directly. For 180 kA magnesium electrolysis cell, the balance point is at 25 W·(m °C)^−1^. For 240 kA electrolysis cell, the balance point is at 26.5 W·(m °C)^−1^. For the 300 kA electrolysis cell, the minimum of the heat balance difference point is at 27.2 W·(m °C)^−1^. Above all, adjusting the anode heat transfer coefficient is an effective means to make the thermal field of the cell in balance.

#### 3.2.5. The effect of other heat transfer coefficients on heat balance

In the case of constant heat transfer area, changing the heat transfer coefficient of the anode head to improve the thermal balance of the cell is an effective means. It is necessary to study the effects of the cell cover and cell shell heat transfer coefficient on heat balance because the heat dissipating capacity of cell cover, cell shell accounts for 40% of the total.

**Figure 15.**
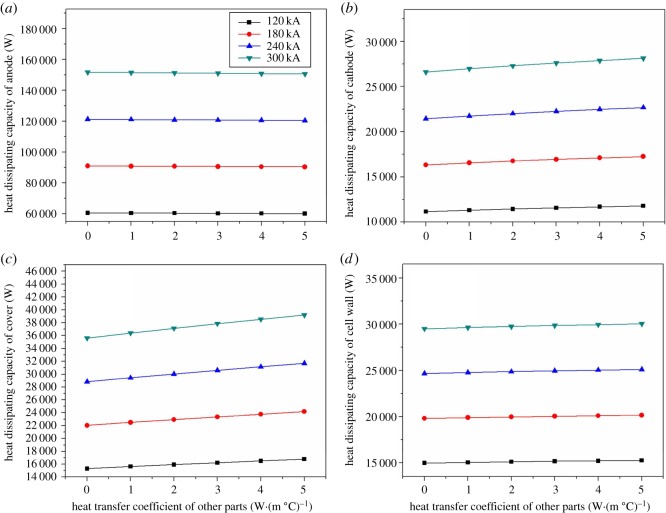
The relationship between heat transfer coefficients of other parts and various parts on heat balance.

Figure 15*a* shows the heat dissipating capacity of the anode head in different current intensity decreases slightly with the heat transfer coefficient of other parts increasing. The minimum drop of the heat dissipating capacity is 420.6 W, and the biggest drop is about 1038.7 W. The reason of this phenomenon is that the heat which is flowing to anode originally has been flowed to other parts with the increase of the heat transfer coefficient of other parts. The heat dissipating capacity of cathode, cell cover and cell shell increases by degrees with the heat transfer coefficient increasing which has been shown in figure 15*b–d*. Among them, the heat dissipating capacity of cell cover changes greatly. With the heat transfer coefficient of 120 kA magnesium electrolysis cell increasing, the heat dissipation of the cathode, cell cover and cell shell increases by 645.3, 1467.9 and 280.4 W respectively. Similarly, the heat dissipation of the cathode, cell cover and cell shell of 180 kA electrolysis cell increases by 947.5, 2149.4 and 354 W, respectively. The result changes into 1231.9, 2866.1 and 448.1 W when the current intensity is 240 kA, and the result changes into 1526.4, 3583.7 and 530.1 W when the current intensity is 300 kA. According to the heat loss of the four types of electrolysis cell, the most obvious growth of heat dissipation capacity comes from cell cover, followed by cathode head, and finally cell shell.

**Figure 16.**
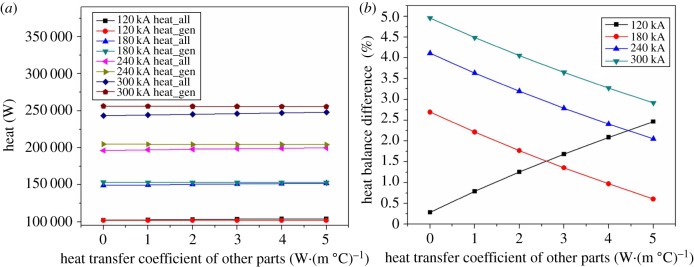
The relationship between heat transfer coefficients of other parts and heat balance difference.

As figure 16 shows, the 120 kA magnesium electrolysis cell increasingly deviates from the heat balance point with the increase of the heat transfer coefficient of other parts, and the other electrolysis cells of different current intensity show the opposite trend. The 180 kA electrolysis cell reaches the heat balance, at about 5 W·(m °C)^−1^, when the heat transfer coefficient increases by about 2 W·(m °C)^−1^, while that of the 240 and 300 kA electrolysis cells still did not reach 2% of difference rates. Comparing with the method which adjusts the heat transfer coefficient of anode, the methods of adjusting the heat transfer coefficient of other parts can be considered next. Although it can meet heat balance to a certain extent, but this way cannot meet all types of magnesium electrolysis cells.

#### 3.2.6. The effect of anode–cathode distance (ACD) on heat balance

The size of ACD not only affects the size of electric potential, but also the area of dissipation. As a result, it will influence the size of heat dissipating capacity, and ultimately the heat balance difference. It is a very important parameter that must be studied deeply during the process of designing a magnesium electrolysis cell.

**Figure 17.**
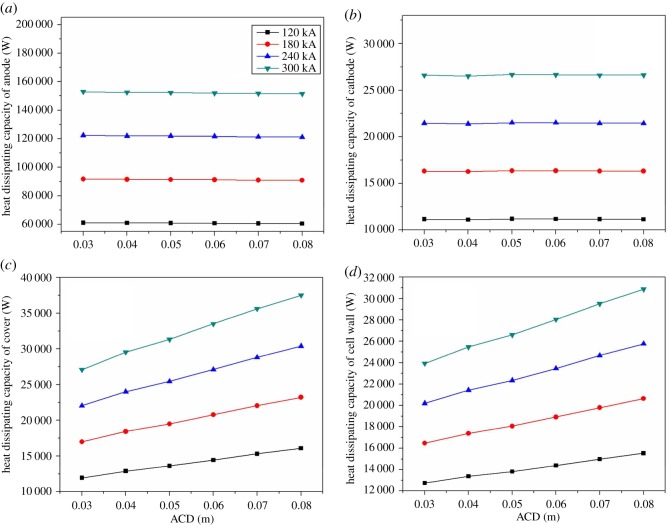
(*a*–*b*) The heat relationship between ACD and various parts.

Figure 17*a,b* show that the heat dissipating capacity of anode and cathode is almost unchanged when the size of ACD is changing. The most obvious change happens on the cell cover and cell shell by adjusting ACD. In figure 17*c,d*, the growth rates of the heat dissipating capacity increase when the current intensity gets larger. The cell cover and cell shell of 300 kA will be more sensitive to the adjustment of ACD. For the 120 kA electrolysis cell, as ACD has grown from 0.03 to 0.08 m, the heat dissipating capacity of cell cover has changed by 4155.8 W, and cell shell has changed by 2801.3 W; for 180 kA cell, the value of cell cover has increased by 6232.4 W, and cell shell has increased by 4173.8 W; for 240 kA cell, the value of cell cover has increased by 8353.6 W, and cell shell has increased by 5571.4 W; for 300 kA cell, the value of cell cover has increased by10 434.3 W, and cell shell has increased by 6938.6 W. Above all, adjusting ACD can take the heat balance of the cell into control. In this study, the reduction of heat dissipation capacity, which is caused by adjusting ACD, accounts for 26%.

**Figure 18.**
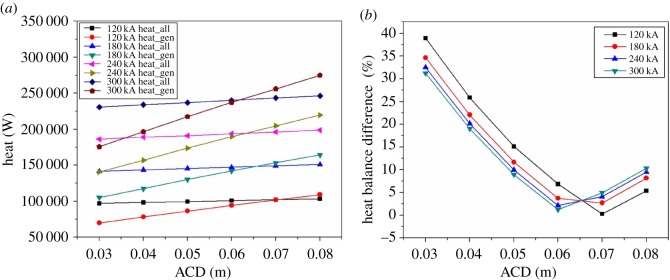
The relationship between ACD and heat balance difference.

As figure 18 shows, for electrolysis cells in all kinds of current intensity, the heat balance point changes with ACD. Furthermore, with the increase of the current intensity, the balance point moves towards the direction that the polar distance decreases. When ACD is bigger than 0.08 m, heat dissipation rate is much higher than the Joule heat production rate. The cell of 120, 180, 240 and 300 kA reaches the heat balance point when ACD is 0.07, 0.0655, 0.063 and 0.062 m, respectively. And the difference between heat generation and heat dissipation of these four types of cell is about 102 126, 148 241, 194 585 and 240 864 W, respectively. From figure 18*b*, it can be observed that the changing trend of heat balance difference of these four types of cell is almost unchanged. In a word, the heat balance in electrolysis cell can be achieved through adjusting ACD which is also an extremely effective way.

**Figure 19.**
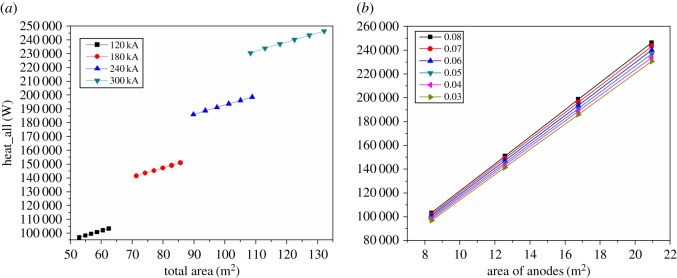
The relationship between areas and heat dissipating capacity.

In figure 19, the total heat dissipation area and the total heat loss was affected by changing ACD in the cells of different current intensity. In figure 19*a*, there are four ladder sections, and the slope of every ladder section maintains basically the same, which explains that the heat dissipation capacity is related to the size of heat dissipation area rather than current intensity.

## 4. Conclusion

The study focuses on electric and thermal fields in electrolysis industry and the conclusions will be of benefit to batteries and electrochemistry. The paper mainly researched the effects of the structure parameters on the thermal field in magnesium electrolysis cells. The influence of current intensity, electrolysis temperature, ambient temperature, heat transfer coefficient and ACD on the heat balance of electrolysis cell is studied by finite-element simulation of actual 120 kA electrolysis cell. Some conclusions which can be obtained by simulation calculation are as follows:

(1) Under the condition of same current intensity, the heat dissipating capacity increases linearly with the 120 kA electrolysis cell amplification to 480 kA in parallel. However, the electrolysis cell deviates from the heat balance increasingly. When it is in 240 kA, heat absorbed will be over 5.6%; when it is in 480 kA, the value will close to 7.9%. Therefore, the simple amplification cannot meet the requirements of heat balance, and the design of the electrolysis cell needs to be further optimized.
(2) With the increase of electrolysis temperature, the growth rate of heat dissipation at anode is the largest, followed by cathode, cell shell and cell cover. The ability of changing electrolysis temperature to keeping the heat balance is limited, and the heat balance difference only reaches about 2%.
(3) The ambient temperature needs to be considered in the design process of the magnesium electrolysis cell. For the 300 kA electrolysis cell, the heat dissipating capacity, which accounts for 3% of the total, increases by 7570.9 W when the ambient temperature drops from 35°C to 15°C.
(4) Commonly, the heat dissipation capacity of anode is about 60% of the total. The simulation results by changing the heat transfer coefficient of anode show that the heat dissipation increases by nearly 9.9% when the anode heat transfer coefficient increases by 5 W·(m °C)^−1^. In a word, adjusting the anode heat transfer coefficient is an effective means to make heat loss in balance of the cell.
(5) In addition to the anode head, the heat dissipation coefficient of other parts of the cell also has an important effect on the heat balance. When the coefficient increases by 5 W·(m °C)^−1^, the total heat dissipation capacity increases by 2.27%. Which means adjusting the heat dissipation coefficient of other parts, such as cell cover and cell shell, can be secondary means in optimizing the magnesium electrolysis cell.
(6) The heat dissipation of cell cover and cell shell is sensitive to the change of ACD. The growth of the heat dissipation of the 300 kA magnesium electrolysis cell is 10 434.5 W when ACD increased by 0.05 m. Thus, changing the ACD is an extremely effective way to achieve the heat balance compared with adjusting the heat transfer coefficient of anode.

## Data accessibility

The paper includes all information. The structural parameters of the electrolysis cell are listed in the table 1, and material properties are listed in tables 2–4.

## References

[1] Bradwell DJ, Kim H, Sirk AHC, Sadoway DR 2012 Magnesium–antimony liquid metal battery for stationary energy storage. J. Am. Chem. Soc. 134, 1895–1897. (doi:10.1021/ja209759s) 22224420 10.1021/ja209759s

[2] Hoopes W 1925 Electrolytically-refined aluminum and articles made therefrom. U.S. Patent 1,534,315.

[3] Shen Y, Zikanov O 2016 Thermal convection in a liquid metal battery. Theor. Comput. Fluid Dyn. 30, 275–294. (doi:10.1007/s00162-015-0378-1)

[4] Köllner T, Boeck T, Schumacher J 2017 Thermal Rayleigh-Marangoni convection in a three-layer liquid-metal-battery model. Phys. Rev. E 95, 11 (doi:10.1103/PhysRevE.95.053114) 10.1103/PhysRevE.95.053114 28618570

[5] Wang W, Wang K 2016 Simulation of thermal properties of the liquid metal batteries. *6th Int. Conf. on Power Electronics Systems and Applications (PESA), Hong Kong, 15–17 December 2015*, pp. 1–11. IEEE (doi:10.1109/PESA.2015.7398882)

[6] Weber N, Galindo V, Stefani F, Weier T 2014 Current-driven flow instabilities in large-scale liquid metal batteries, and how to tame them. J. Power Sources 265, 166–173. (doi:10.1016/j.jpowsour.2014.03.055)

[7] Weber N, Galindo V, Weier T, Wondark T 2015 Simulation of instabilities in liquid metal batteries. Direct and Large-Eddy Simul. IX. 20, 585–591.

[8] Stefani F, Weier T, Gundrum T, Gerbeth G 2011 How to circumvent the size limitation of liquid metal batteries due to the Tayler instability. Energy Convers. Manage. 52, 2982–2986. (doi:10.1016/j.enconman.2011.03.003)

[9] Weber N, Beckstein P, Herreman W, Horstmann GM, Nore C, Stefani F, Weier T 2017 Sloshing instability and electrolyte layer rupture in liquid metal batteries. Phys. Fluids 29, 054101–051534. (doi:10.1063/1.4982900)

[10] Morillo A, Freund A, Merten C 2004 Concept and design of a novel compact reactor for autothermal steam reforming with integrated evaporation and CO cleanup. Indust. Eng. Chem. Res. 43, 4624–4634. (doi:10.1021/ie0341449)

[11] Shahrokhi M, Nejati A 2002 Optimal temperature control of a propane thermal cracking reactor. Indust. Eng. Chem. Res. 41, 6572–6578. (doi:10.1021/ie0106783)

[12] Zhou NJ, Mei Z, Jiang C, Zhou P, Li J 2003 Coupled computation method of physics fields in aluminum reduction cells. T. Nonferr. Metal Soc. 13, 431–437.

[13] Dupuis M, Fradet C 1998 Using ANSYS® based aluminum reduction cell energy balance models to assist efforts to increase Lauralco's smelter productivity. Proc. ANSYS 8th Int. Conf. Pittsburgh, PA, January 2, 233–240.

[14] Eklund H, Engseth PB, Langseth B, Mellerud T, Wallevik O 2016 An improved process for the production of magnesium. In Essential readings in magnesium technology (eds SN Mathaudhu, AA Luo, NR Neelameggham, EA Nyberg, WH Sillekens), pp. 141–144. Cham, Switzerland: Springer (doi:10.1007/978-3-319-48099-2_23)

[15] Haupin WE 1971 Calculating thickness of containing walls frozen from melt. JOM 23, 41–44. (doi:10.1007/BF03355715)

[16] Kryukowsky V 1992 Mathematical modelling of heat transfer in pots lining materials for production of nonferrous metals. Light Met. 1992, 557–562.

[17] Pfundt H, Vogelsang D, Gerling U 1989 Calculation of the crust profile in aluminum reduction cells by thermal computer modelling. Light Met. 1989, 371–377.

[18] Valles A, Lenis V, Rao M 1995 Prediction of ledge profile in Hall-Heroult cell. Light Met. 1995, 309–313.

[19] Peacey JG, Medlin GW 1979 Cell sidewall studies at Noranda aluminum. Light Met. 1979, 475–492.

[20] Ek A, Fladmark GE 2013 Simulation of thermal, electric and chemical behaviour of an aluminum cell on a digital computer. *Essential Readings in Light Metals: Aluminum Reduction Technology*, Vol. 2 (eds G Bearne, M Dupuis, G Tarcy), ch. 39. Hoboken, NJ: John Wiley (doi:10.1002/9781118647851.ch39)

[21] Tomasino T, Martin C, Waz E 2004 Numerical modeling of heat transfer around an aluminum reduction pot shell. Light Met. 2004, 433–438.

[22] Dupuis M 2000 Using ANSYS to model aluminum reduction cell since 1984 and beyond. Light Met. 2000, 307–313.

[23] Dupuis M 2000 Development of a 3D transient thermo-electric cathode panel erosion model of an aluminum reduction cell. Light Met. 2000, 169–178.

[24] Dupuis M, Bojarevics V, Richard D 2008 Impact of the vertical potshell deformation on the MHD cell stability behavior of a 500 kA aluminum electrolysis cell. Light Met. 2008, 409–412.

[25] Dupuis M 1998 Computation of aluminum reduction cell energy balance using ANSYS finite element models. Light Met. 1998, 409–417.

[26] Dupuis M 2011 Development and application of an ANSYS based thermos-electro-mechanical collector bar slot design tool. Light Met. 2011, 517–524.

[27] Dupuis M, Bojarevics V 2005 Weakly coupled thermo-electric and MHD mathematical models of an aluminum electrolysis cell. Light Met. 2005, 449–454.

[28] Mounir B, Seyed MT, Donald Z 2017 LES turbulence modeling approach for molten aluminium and electrolyte flow in aluminum electrolysis cell. Light Met. 2017, 679–686.

[29] Mei Q, Schoenmaker W, Weng SH, Zhuang H, Cheng CK, Chen Q 2016 An efficient transient electro-thermal simulation framework for power integrated circuits. IEEE Trans. Comput. Aided Des. Integr. Circuits Syst. 35, 832–843. (doi:10.1109/TCAD.2015.2488494)

[30] Shcherbinin SA, Fazylov AR, Yakovleva GA 1997 Mathematical simulation of three-dimensional heat and electric fields of magnesium electrolyzer with top anode introduction. Russ. J. Nonferr. Met. 38, 74–76.

[31] Sun Z, Zhang HN, Li P, Li B, Lu GM, Yu JG 2009 Modeling and simulation of the flow field in the electrolysis of magnesium. JOM. 61, 29–33. (doi:10.1007/s11837-009-0066-y)

[32] Sun Z, Li P, Lu GM, Li B, Wang J, Yu JG 2010 Effect of electromagnetic field on three-phase flow behavior. Indust. Eng. Chem. Res. 49, 10 798–10 803. (doi:10.1021/ie100513w)

[33] Sun Z, Zhao Y, Lu GM, Li P, Wang J, Yu JG 2011 Novel method based on electric field simulation and optimization for designing an energy-saving magnesium electrolysis cell. Indust. Eng. Chem. Res. 50, 6161–6173. (doi:10.1021/ie101091p)

[34] Liu CL, Sun Z, Lu GM, Song XF, Yu JG 2014 Scale-up design of a 300 kA magnesium electrolysis cell based on thermo-electric mathematical models. Can. J. Chem. Eng. 92, 1197–1206. (doi:10.1002/cjce.21940)

[35] Zhang YJ 2006 Electrolytic metallurgy of magnesium. Changsha, People's Republic of China: Central South University Press.

[36] Yang S, Zhang HL, Zou Z, Lai YQ, Li J 2015 Calculation of heat transfer coefficient between aluminum reduction cell surface and surroundings. Chi. J. Nonferr. Metals. 25, 515–522.

